# The influence of genetic polymorphisms on cytokine profiles in pediatric COVID-19: a pilot study

**DOI:** 10.3389/fped.2025.1523627

**Published:** 2025-02-24

**Authors:** Kateryna Kozak, Halyna Pavlyshyn, Oleksandr Kamyshnyi, Oksana Shevchuk, Mykhaylo Korda, Sandor G. Vari

**Affiliations:** ^1^ Department of Pediatrics No. 2, I. Horbachevsky Ternopil National Medical University, Ternopil, Ukraine; ^2^ Department of Microbiology, Virology, and Immunology, I. Horbachevsky Ternopil National Medical University, Ternopil, Ukraine; ^3^ Department of Pharmacology and Clinical Pharmacology, I. Horbachevsky Ternopil National Medical University, Ternopil, Ukraine; ^4^ Department of Medical Biochemistry, I. Horbachevsky Ternopil National Medical University, Ternopil, Ukraine; ^5^ International Research and Innovation in Medicine Program, Cedars–Sinai Medical Center, Los Angeles, CA, United States

**Keywords:** genetic polymorphism, cytokine, COVID-19, children, immune response

## Abstract

**Introduction:**

Recent studies have underscored the importance of genetic factors in predicting COVID-19 susceptibility and severity. While cytokine storms are crucial in disease severity, genetic predisposition significantly influences immune responses. Our study examined genes related to SARS-CoV-2 invasion *(ACE2 rs2074192*) and interferon-induced immunity (*IFNAR2 rs2236757, TYK2 rs2304256, OAS1 rs10774671, OAS3 rs10735079*). Additionally, we investigated genes linked to Kawasaki disease (*CD40 rs4813003, FCGR2A rs1801274, CASP3 rs113420705*) that play roles in immunogenesis.

**Methods:**

The pilot study, which involved 75 pediatric patients aged one month to 17 years [43 patients with active COVID-19, 17 children with multisystem inflammatory syndrome in children (MIS-C), and 15 healthy controls], was conducted in Ternopil, Ukraine. Gene polymorphism was studied for all patients. ELISA kits were used for interleukin studies, including Human IL-1β (Interleukin 1 Beta), Human IL-6 (Interleukin 6), Human IL-8 (Interleukin 8), Human IL-12 (Interleukin 12), Human IFN-α (Interferon Alpha), and Human TNF-α (Tumor Necrosis Factor Alpha). Statistical analysis was performed using IBM SPSS Statistics 21 and GraphPad Prism 8.4.3.

**Results:**

The analysis identified significant gene-cytokine associations in pediatric COVID-19 patients. The *ACE2 rs2074192* T allele correlated with increased IL-1β, IL-6, IL-8, and TNF-α. The *IFNAR2 rs2236757* A allele was linked to elevated IL-1β and IL-12 levels and low IFN-α levels, while *OAS1 rs10774671* A allele carriers also exhibited lower IFN-α levels. *OAS1 rs10774671* was prognostically crucial for determining IL-8 levels in children infected with SARS-CoV-2. *OAS3* gene polymorphism *rs10735079* was associated with changes in IL-6 levels, precisely a high level. The *CD40 rs4813003* T allele increased IFN-α levels, while carriers of allele C had higher levels of IL-12. The results of our study revealed a correlation between IL-8 levels and the *FCGR2A* gene polymorphism *rs1801274* (A/G). The *CASP3* gene polymorphism *rs113420705* led to an increase in IL-6.

**Conclusion:**

These findings enhance our understanding of pediatric COVID-19 and may hold promise for developing targeted interventions and providing a personalized medical approach for each patient.

## Introduction

Recent studies have highlighted the role of various genes, genotypes, and alleles in predicting COVID-19 susceptibility, severity, and mortality in both pediatric and adult populations. These include *ACE2, ACE1, AGT, DDR1, IFITM3, IFNL4, HLA-A, -B, -C, -E, -DRB1, IFNL3, IL-6, IL-10, ABO, CCR5, APOE, PNPLA3, TLL1, S1R, MBL2, FPR1, DPP4, TMPRSS2, KLRC2, VDR* and many others (1–5). Previously, we have published and demonstrated the specific roles of the ACE2 rs2074192, IFNAR2 rs2236757, OAS1 rs10774671, CD40 rs4813003, and *CASP3 rs113420705* genes in predicting severe COVID-19 and multisystem inflammatory syndrome (MIS-C) in children (2). Although the cytokine storm is a crucial factor influencing COVID-19 severity, genetic predisposition remains a critical determinant of the immune response.

The body's immune response is triggered when the SARS-CoV-2 virus enters a cell and interacts with the ACE2 receptor and Transmembrane protease, serine 2 (TMPRSS2). This leads to the activation of Th1, which stimulates the secretion of pro-inflammatory cytokines such as interleukin-6 and granulocyte-macrophage colony-stimulating factor (GM-CSF) (6). It should be emphasized that GM-CSF also stimulates the secretion of other pro-inflammatory cytokines, including IL-6, TNF-α, IL-23, and CCL17, by monocytes and macrophages (7). The pathogenesis of the cytokine storm is also linked to angiotensin II/angiotensin receptor (AT1R)-induced hypercytokinemia and hyperinflammatory syndrome (6, 8). Studies have shown that SARS-CoV-2 activates the NF-kappa-B (NF-κB) nuclear transcription factor through pattern-recognition receptors (PPR). This leads to a decreased ACE2 expression, increasing angiotensin II levels. The angiotensin II/angiotensin type 1 receptor axis, through disintegrin and metalloproteinase with thrombospondin motif 17 (ADAM17), also stimulates the secretion of TNF-α and the soluble form of IL-6Ra (sIL-6Ra) (6, 9). IL-6, when bound to sIL-6Ra, activates the Signal transducer and activator of transcription-3 (STAT3), which then induces their expression by binding to the promoters of genes responsible for inflammation (6, 10). STAT3 activation is also associated with the polarisation of macrophages into pro-inflammatory (M1) and anti-inflammatory (M2) types. Activated macrophages can produce pro-inflammatory cytokines such as TNF-α, IL-1, IL-6, and IL-18, which can trigger the cascade of a cascade of pro-inflammatory markers and ultimately lead to a cytokine storm. Current researches have highlighted the role of pro-inflammatory cytokines (IL-1β, IL-2, IL-6, IL-7, IL-8, IL-9, IL-10, IL-17, IL-18, IL-22, IL-33, TNF-α, IFN-γ, IFN-γ induced protein 10 (IP-10), monocyte chemoattractant protein-1 (MCP-1), and granulocyte-macrophage colony-stimulating factor (GM-CSF), chemokine (C-X-C motif) ligand (CXCL)10) in the severity of COVID-19 (11–15). A meta-analysis of 77 prospective or retrospective cohort studies in adult patients with COVID-19 showed that IL-1β, IL-2R, IL-4, IL-6, IL-8, IL-10, and IL-17, inflammatory markers, were potential risk factors for severe SARS-CoV-2 infection or mortality. The analysis was conducted before therapy initiation (16). Kang et al. also demonstrated higher levels of IL-6 and IL-8 in adults with severe COVID-19 courses compared to non-severe patients (17). Recent studies in children with MIS-C have also highlighted the significantly higher levels of IL-1 receptor antagonist, IL-1β, IL-8, IL-10, IL-6, IL-27, and TNF-α in patients with MIS-C compared to health control (18, 19). Notably, Talariko et al. did not find a significant difference in IL-6 levels between pediatric patients with asymptomatic or mild COVID-19 and those with moderate or severe disease progression, suggesting the need to identify other factors related to disease severity (20).

Recent researches on SARS-CoV-2 infection has documented factors that contribute to cytokine storm and the severity of COVID-19. These factors include age, male sex, and comorbid conditions such as hypertension, congenital heart disease, diabetes, obesity, chronic lung, liver and kidney diseases, cerebral palsy, epilepsy, sickle cell disease, cancer and chemotherapy, immunodeficiency, and different host genetic backgrounds (21–25). However, most of studies have primarily focused on genes such as *ACE2* and *TMPRSS2*, which are responsible for the entry and replication of SARS-CoV-2, and the signaling pathways triggered by the virus in the adults (24, 26). Further insights can improve our understanding of COVID-19 pathogenesis in the pediatric population. In our study, we specifically examined genes involved in the process of SARS-CoV-2 invasion into the cell (*ACE2 rs2074192*), as well as genes associated with interferon-induced immune response (*IFNAR2 rs2236757, TYK2 rs2304256, OAS1 rs10774671, OAS3 rs10735079*). We also investigated several genes that are known to be associated with Kawasaki disease and play a crucial role in immunogenesis (*CD40 rs4813003* and *FCGR2A rs1801274*, which are critical in regulating B-cell immune response, and *CASP3 rs113420705*, a gene associated with apoptosis, which is extremely important in viral infection).

## Materials and methods

The pilot study included 75 pediatric patients aged one month to 17 years: 43 patients with active COVID-19, 17 children with multisystem inflammatory syndrome in children (MIS-C), and 15 healthy children without evidence of COVID-19 (SARS-CoV-2 PCR negative and IgG negative). Among the 43 patients with COVID-19, 20 children had a mild course of the disease, 10 had moderate disease severity, and 13 experienced severe COVID-19. MIS-C was diagnosed according to the World Health Organization's criteria (27).

The research was conducted in Ternopil, Ukraine, across two pediatric hospital settings: Ternopil Municipal Children's Hospital and Ternopil Regional Children's Clinical Hospital. Informed consent was obtained from all children's caregivers and the Bioethics Committee of I. Horbachevsky Ternopil National Medical University (Protocol No. 71, dated October 25, 2022) approved the study.

Gene polymorphism was studied for all patients. We focused on eight immunoregulatory genes−*ACE2, IFNAR2, TYK2, OAS1, OAS3, CD40, FCGR2A,* and *CASP3*. Genomic DNA was extracted using the Thermo Scientific™ GeneJET™ Whole Blood Genomic DNA Purification Mini Kit (Cat. No. K0781, Thermo Fisher Scientific, Waltham, MA, USA). DNA was amplified using the TaqMan™ Universal Master Mix II, no UNG (5 ml, Cat. No. 4440040) with real-time polymerase chain reaction (PCR). For genotyping, predesigned TaqMan™ SNP Genotyping Assays (Cat. No. 4351379, Thermo Fisher Scientific, Waltham, MA, USA) were employed to analyze the following SNPs: *ACE2 rs2074192, IFNAR2 rs2236757, TYK2 rs2304256, OAS1 rs10774671, OAS3 rs10735079, CD40 rs4813003, FCGR2A rs1801274*, and *CASP3 rs113420705*.

The current study uses data from the same cohort previously assessed in a survey of the relationship between COVID-19 severity in children and immunoregulatory gene polymorphism (2). The data from this cohort were further analyzed to investigate the interleukin profile in pediatric patients with COVID-19 and MIS-C.

Venous blood samples were collected on the first day of hospital admission before initiating therapy. In case of MIS-C low-dose corticosteroids or infusion of intravenous immune globulin were not used before sample collection. The cytokines were measured from plasma without any prior cellular stimulation. ELISA kits were used for interleukin studies, including Human IL-1β (Interleukin 1 Beta) (Cat.No.:E-EL-H0149); Human IL-6 (Interleukin 6) (Cat.No.:E-EL-H6156); Human IL-8 (Interleukin 8) (Cat.No.:E-EL-H6008); Human IL-12 (Interleukin 12) (Cat.No.:E-EL-H0150); Human IFNα (Interferon Alpha) (Cat.No.:E-EL-H6125); Human TNF-α (Tumor Necrosis Factor Alpha) (Cat.No.:E-EL-H0109).

G*Power 3.1.9.7 was used to calculate the required sample size. An exact test for linear multiple regression was performed with the following input parameters: two-tailed test; H1 *ρ*^2^ = 0.3 (indicating a moderate effect size); H0 *ρ*^2^ = zero (no effect); *α* error probability = 0.05; power (1—β error probability) = 0.95; and number of predictors = 8. The output parameters indicated that the total sample size needed for our study was 74.

Statistical analysis was performed using IBM SPSS Statistics 21 and GraphPad Prism 8.4.3. Numerical data had a non-normal distribution; therefore, the data were presented as the median and interquartile range. Two independent samples were compared using the Mann–Whitney *U* test, and the Kruskal–Wallis ANOVA test was used for three variables. The two-tailed Fisher's exact test was utilized for 2 × 2 contingency tables, while Pearson's Chi-square test was employed for 3 × 2 tables. In situations where the data in a 3 × 2 contingency table did not meet the necessary criteria for Pearson's chi-squared test (i.e., expected frequencies of at least 1 for each cell and expected frequencies of at least 5 for 80% of the cells), the Fisher's exact test was used. Linear regression assessed the most predictive values for interleukin level determination. In regression analysis next values were assessed: R; R-Squared (*R*^2^); Adjusted R-Squared; Standard Error of the Estimate; F-Statistic; *p*-Value for the Model. Results were considered significant at a *p*-value of <0.05.

## Results

The demographic characteristics of the patients involved in the study are presented in Table 1. The age of patients diagnosed with MIS-C was significantly higher than the age of children diagnosed with COVID-19. However, the sex distribution among the three study groups was not significantly different. Additionally, patients with MIS-C had a longer duration of illness before blood sampling compared to those with COVID-19. Notably, the incidence of comorbidities was similar between patients with acute SARS-CoV-2 and MIS-C infections. Some of the comorbidities observed in children with COVID-19 included obesity, congenital heart disease (complete atrioventricular septal defect), left-sided congenital renal aplasia, and Epstein–Barr virus-associated hepatitis. In two patients with MIS-C, there were also other underlying conditions present, such as congenital cerebral defect (cerebral atrophy of the frontal, insular, and temporal lobes) and juvenile rheumatoid arthritis.

**Table 1:** Demographic characteristic of the pediatric patients with COVID-19 and MIS-C and healthy children involved in the study.

Group	Age, years	Sex, *n* (%)		Disease duration before hospitalization, days	Presence of comorbidities, *n* (%)
		Male	Female		
COVID-19	3.00 (1.00; 11.00)	26 (60.47)	13 (76.47)	2 (1; 5)	4 (9.30)
MIS-C	7.00 (2.90; 11.80)	17 (39.53)	4 (23.53)	4 (4; 6)	2 (11.76)
Control	8.00 (5.00; 13.00)	7 (46.67)	8 (53.33)	−	−
	H = 7.60; *p* = 0.022^a^	*χ*^2^ = 3.02; *p* = 0.221		*p* = 0.021^a^	р = 1.000

^a^ Statistical significance.

The genetic characteristics of the children included in the research are presented in Tables 2, 3. The study found no significant differences in gene polymorphism between patients with COVID-19 and MIS-C. However, the genetic composition of the main study group (patients infected with SARS-CoV-2) differed from that of the control group. Specifically, the results showed a higher prevalence of the AA genotype *OAS1 rs10774671* and the CC genotype *CD40 rs4813003*, as well as a lower frequency of the TT genotype *CASP3 rs113420705* among patients with SARS-CoV-2 infection compared to healthy children. Furthemore, there was a statistically significant difference in the frequency of alleles, with the A allele of *OAS1 rs10774671* and the C allele of *CD40 rs4813003* being more prevalent in the group of children infected with SARS-CoV-2.

**Table 2:** Genotype profiles in SARS-CoV-2 infected and healthy pediatric patients.

Gene	Genotype	COVID-19	MIS-C	Healthy control	*χ*^2^; *p*	SARS-CoV-2 (total)	Healthy control	χ^2^; *p*
*ACE2 rs2074192*	CC	15 (34.88)	6 (35.29)	8 (53.33)	*χ*^2^ = 3.12; *p* = 0.538	21 (35.00)	8 (53.33)	*χ*^2^ = 2.87; *p* = 0.239
	CT	10 (23.26)	3 (17.65)	4 (26.67)		13 (21.67)	4 (26.67)	
	TT	18 (41.86)	8 (47.06)	3 (20.00)		26 (43.33)	3 (20.00)	
*IFNAR2 rs2236757*	GG	14 (32.56)	4 (23.53)	7 (46.67)	*χ*^2^ = 6.36; *p* = 0.174	18 (30.00)	7 (46.67)	*χ*^2^ = 2.92; *p* = 0.232
	GA	12 (27.91)	9 (52.94)	6 (40.00)		21 (35.00)	6 (40.00)	
	AA	17 (39.53)	4 (23.53)	2 (13.33)		21 (35.00)	2 (13.33)	
*TYK2 rs2304256*	CC	20 (46.51)	8 (47.06)	9 (60.00)	*χ*^2^ = 1.14 *p* = 0.566	28 (46.67)	9 (60.00)	*χ*^2^ = 1.14; *p* = 0.566
	CA	16 (37.21)	7 (41.18)	5 (33.33)		23 (38.33)	5 (33.33)	
	AA	7 (16.28)	2 (11.76)	1 (6.67)		9 (15.00)	1 (6.67)	
*OAS1 rs10774671*	GG	12 (27.19)	1 (5.88)	8 (53.33)	*χ*^2^ = 12.98; *p* = 0.011^a^	13 (21.67)	8 (53.33)	*χ*^2^ = 9.58; *p* = 0.008^a^
	GA	16 (37.21)	10 (58.82)	7 (46.67)		26 (43.33)	7 (46.67)	
	AA	15 (34.88)	6 (35.29)	0		21 (35.00)	0	
*OAS3 rs10735079*	AA	21 (48.84)	7 (41.18)	10 (66.67)	*χ*^2^ = 2.32; *p* = 0.678	28 (46.67)	10 (66.67)	*χ*^2^ = 1.94; *p* = 0.379
	AG	12 (27.91)	6 (35.29)	3 (20.00)		18 (30.00)	3 (20.00)	
	GG	10 (23.26)	4 (23.53)	2 (13.33)		14 (23.33)	2 (13.33)	
*CD40 rs4813003*	CC	38 (88.37)	15 (88.24)	10 (66.67)	*χ*^2^ = 6.51; *p* = 0.164	53 (88.33)	10 (66.67)	*χ*^2^ = 6.51; *p* = 0.039^a^
	CT	5 (11.63)	2 (11.76)	4 (26.67)		7 (11.67)	4 (26.67)	
	TT	0	0	1 (6.67)		0	1 (6.67)	
*FCGR2A rs1801274*	AA	16 (37.12)	6 (35.29)	8 (53.33)	χ^2^ = 1.91; *p* = 0.753	22 (36.67)	8 (53.33)	*χ*^2^ = 1.63; *p* = 0.442
	AG	17 (39.53)	6 (35.29)	5 (33.33)		23 (38.33)	5 (33.33)	
	GG	10 (23.26)	5 (29.41)	2 (13.33)		15 (25.00)	2 (13.33)	
*CASP3 rs113420705*	TT	3 (6.98)	3 (17.65)	7 (46.67)	*χ*^2^ = 12.31; *p* = 0.015^a^	6 (10.00)	7 (46.67)	*χ*^2^ = 11.32; *p* = 0.003^a^
	TC	32 (74.42)	11 (64.71)	6 (40.00)		43 (71.67)	6 (40.00)	
	CC	8 (18.60)	3 (17.65)	2 (13.33)		11 (18.33)	2 (13.33)	

^a^ Statistical significance.

**Table 3:** Allelic profiles in SARS-CoV-2 infected and healthy pediatric patients.

Gene	Allele	COVID-19	MIS-C	Healthy control	*χ*^2^; *p*	SARS-CoV-2 (total)	Healthy control	*p*
*ACE2 rs2074192*	C	40 (46.51)	15 (44.12)	20 (66.67)	*χ*^2^ = 4.22; *p* = 0.121	55 (45.83)	20 (66.67)	*p* = 0.065
	T	46 (53.49)	19 (55.88)	10 (33.33)		65 (54.17)	10 (33.33)	
*IFNAR2 rs2236757*	G	40 (46.51)	17 (50.00)	20 (66.67)	*χ*^2^ = 3.65; *p* = 0.161	57 (47.50)	20 (66.67)	*p* = 0.069
	A	46 (53.94)	17 (50.00)	10 (33.33)		63 (52.50)	10 (33.33)	
*TYK2 rs2304256*	C	56 (65.12)	23 (67.65)	23 (76.67)	*χ*^2^ = 1.37; *p* = 0.505	79 (65.83)	23 (76.67)	*p* = 0.283
	A	30 (34.88)	11 (32.35)	7 (23.33)		41 (34.17)	7 (23.33)	
*OAS1 rs10774671*	G	40 (46.51)	12 (35.29)	23 (76.67)	*χ*^2^ = 11.89; *p* = 0.003^a^	52 (43.33)	23 (76.67)	*p* = 0.002^a^
	A	46 (53.49)	22 (64.71)	7 (23.33)		68 (56.67)	7 (23.33)	
*OAS3 rs10735079*	A	54 (62.79)	20 (58.82)	23 (76.67)	*χ*^2^ = 2.53; *p* = 0.282	74 (61.67)	23 (76.67)	*p* = 0.140
	G	32 (37.21)	14 (41.18)	7 (23.33)		46 (38.33)	7 (23.33)	
*CD40 rs4813003*	C	81 (94.19)	32 (94.12)	24 (80.00)	*χ*^2^ = 6.09; *p* = 0.048^a^	113 (94.17)	24 (80.00)	*p* = 0.024^a^
	T	5 (5.81)	2 (5.88)	6 (20.00)		7 (5.83)	6 (20.00)	
*FCGR2A rs1801274*	A	49 (56.98)	18 (52.94)	21 (70.00)	*χ*^2^ = 2.15; *p* = 0.341	67 (55.83)	21 (70.00)	*p* = 0.214
	G	37 (43.02)	16 (47.06)	9 (30.00)		53 (44.17)	9 (30.00)	
*CASP3 rs113420705*	T	37 (43.02)	18 (52.94)	20 (66.67)	*χ*^2^ = 5.13; *p* = 0.077	55 (45.83)	20 (66.67)	*p* = 0.065
	C	49 (56.98)	16 (47.06)	10 (33.33)		65 (54.17)	10 (33.33)	

^a^ Statistical significance.

The interleukin profile of the study groups can be found in Table 4. It is important to note that patients with MIS-C had significantly higher levels of IL-1β, IL-6, IL-8, IL-12, and TNF-α compared to the control group. In contrast, patients with acute SARS-CoV-2 infection only showed elevated levels of IL-6 and IL-8 compared to the control group. Additionally, both main study groups had significantly lower levels of IFN-α compared to healthy individuals. Furthermore, it should be noted that patients with MIS-C had significantly higher levels of IL-1β, IL-6, IL-12, and TNF-α compared to patients with COVID-19.

**Table 4:** Interleukin profile in SARS-CoV-2 infected pediatric patients and healthy controls.

Group		IL-1β	IL-6	IL-8	IL-12	TNF-α	IFN-α
COVID-19	1	3.74 (1.57; 9.67)	74.55 (34.63; 146.71)	14.46 (10.45; 23.62)	21.56 (11.42; 44.94)	52.50 (18.03; 100.34)	37.53 (25.09; 78.58)
MIS-C	2	10.19 (7.86; 15.19)	325.50 (122.90; 504.20)	27.53 (13.93; 53.53)	66.63 (24.61; 178.30)	126.10 (102.80; 528.50)	22.89 (16.68; 29.09)
Control	3	2.24 (0.89; 2.57)	31.56 (19.34; 46.10)	7.68 (4.41; 8.56)	13.31 (10.50; 18.11)	23.54 (16.13; 34.27)	154.10 (68.73; 299.10)
H, *p*		H = 20.11; *p* < 0.001^a^	H = 25.80; *p* < 0.001^a^	H = 17.34; *p* < 0.001^a^	H = 12.10; *p* = 0.002^a^	H = 21.35; *p* < 0.001^a^	H = 26.96; *p* < 0.001^a^
Multiple comparisons		p_1−2_ = 0.004^a^ p_1−3_ = 0.076 p_2−3_ < 0.001^a^	p_1−2_ = 0.001^a^ p_1−3_ = 0.034^a^ p_2−3_ < 0.001^a^	p_1−2_ = 0.061 p_1−3_ = 0.011^a^ p_2−3_ < 0.001^a^	p_1−2_ = 0.025^a^ p_1−3_ = 0.322 p_2−3_ = 0.003^a^	p_1−2_ = 0.001^a^ p_1−3_ = 0.144 p_2−3_ < 0.001^a^	p_1−2_ = 0.077 p_1−3_ < 0.001^a^ p_2−3_ < 0.001^a^

^a^ Statistical significance.

**Figure 1:**
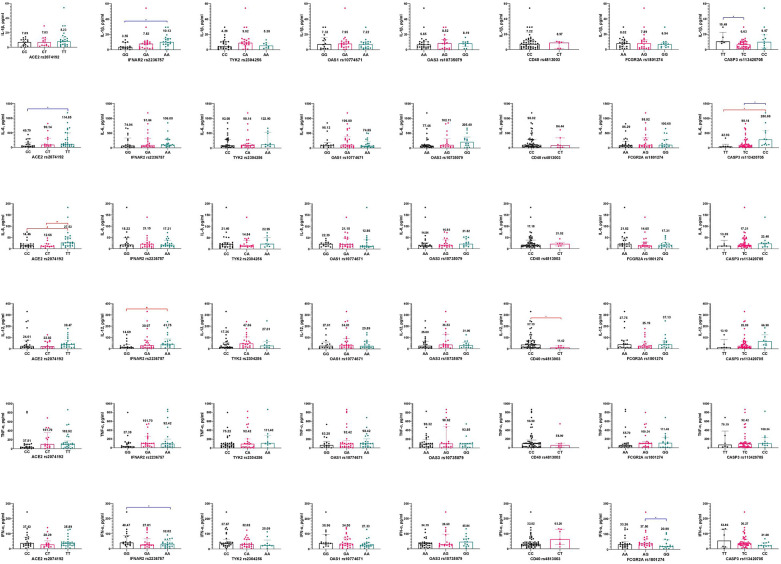
Interleukins (IL-1β, IL-6, IL-8, IL-12, TNF-α, INF-α) levels (pg/ml) in relation to genetic polymorphisms in pediatric patients, infected with SARS-CoV-2. Notes. 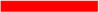
—a significant difference in two groups comparison (adjusted for multiple testing). 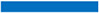
—a significant difference in two groups comparison (unadjusted for multiple testing).

Current research did not reveal any significant changes in IL-1β, TNF-α, and IFN-α levels depending on the studied genotypes when multiple groups were compared (Figure 1) (*p* > 0.05). However, when analyzing the GG and AA genotypes of *IFNAR2 rs2236757*, it was observed that patients with the AA genotype had higher levels of IL-β. Additionally, pediatric patients with the CC genotype of *CASP3 rs113420705* had significantly higher levels of IL-6 compared to those with the TT and TC genotypes (*p* < 0.05). Furthermore, a comparison between patients with CC and TT genotypes of *ACE2 rs2074192* showed that those with the TT genotype had higher levels of IL-6. IL-8 level varies considerably between patients with different *ACE2 rs2074192* polymorphisms—the highest level was registered among children with TT genotype. Notably, IL-12 levels were influenced by the *IFNAR2 rs2236757* and *CD40 rs4813003* polymorphisms. IL-12 levels were higher in patients with the rare homozygous AA genotype (*IFNAR2 rs2236757*) compared to those with the common homozygous GG genotype−41.75 pg/ml and 14.69 pg/ml, respectively. At the same time, the IFN-α level was significantly lower in patients with the AA genotype compared to those with the GG genotype (*IFNAR2 rs2236757*), as well as in patients with the GG genotype (*FCGR2A rs1801274*) compared to those with the AA genotype.

**Figure 2:**
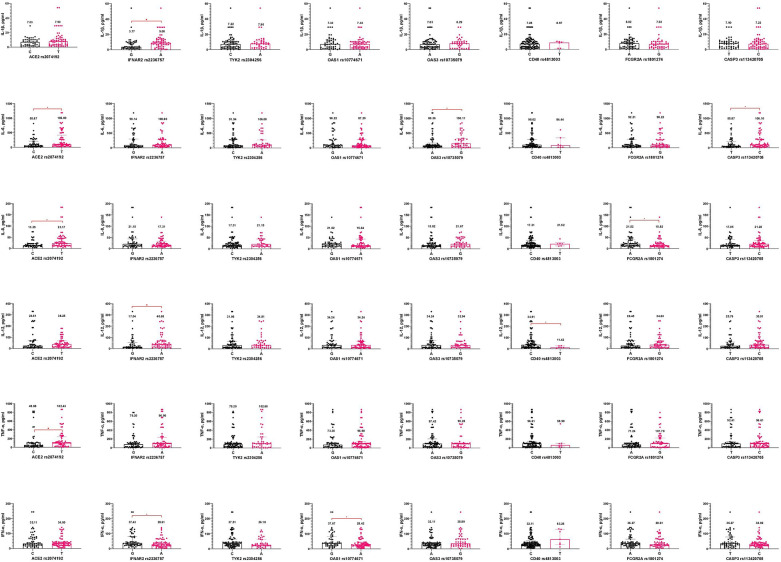
Interleukin concentrations (pg/ml) based on gene alleles in pediatric patients, infected with SARS-CoV-2. Notes. 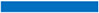
—a significant difference in two groups comparison.

It is important to emphasize that carriers of the T allele of *ACE2 rs2074192* had significantly higher levels of IL-6, IL-8, and TNF-α than carriers of the C allele. Additionally, the A allele in *IFNAR2 rs2236757* was associated with increased IL-1β and IL-12 but decreased IFN-α concentrations in pediatric COVID-19 cases. Changes in IFN-α levels were observed depending on the *OAS1 rs10774671* polymorphism, with carriers of the A allele having lower IFN-α levels than those with the G allele. The G allele of *OAS3 rs10735079* predicted higher IL-6 levels than the A allele. Furthermore, the C allele in *CD40 rs4813003* was associated with significantly higher IL-12 levels. The *FCGR2A rs1801274* gene also influenced IL-8 levels, with patients carrying the A allele exhibiting higher IL-8 levels. Carriers of allele C *CASP3 rs113420705* had higher IL-6 levels than allele T carriers (Figure 2).

Tables 5, 6 present the results of multiple linear regression models evaluating the influence of specific gene alleles on IL-1β, IL-6, IL-8, IL-12, TNF-α, and IFN-α levels. Table 7 summarizes all six models. The IFN-α models showed statistically significant results, indicating that the gene alleles have a meaningful impact on IFN-α level. However, the models for IL-1β, IL-6, IL-8, IL-12, and TNF-α did not reach statistical significance (Table 7).

**Table 5:** Impact of gene alleles on IL-1β, IL-6 and IL-8 levels: regression results.

Predictors	Unstandardized coefficients	Standardized coefficients		t	*p*	95% confidence interval for B	
	B	Std. error	Beta			Lower bound	Upper bound
Model 1 for IL-1β levels							
(Constant)	4.65	6.10		0.76	0.448	−7.46	16.76
ACE2 rs2074192 (C/T)	3.92	1.87	0.21	2.10	0.039^a^	0.21	7.64
IFNAR2 rs2236757 (G/A)	5.01	2.05	0.27	2.44	0.017^a^	0.94	9.09
TYK2 rs2304256 (C/A)	−0.77	2.04	−0.04	−0.38	0.708	−4.82	3.29
OAS1 rs10774671 (G/A)	−1.22	1.88	−0.07	−0.65	0.517	−4.96	2.51
OAS3 rs10735079 (A/G)	−1.91	1.99	−0.10	−0.96	0.338	−5.85	2.03
CD40 rs4813003 (C/T)	−3.71	4.01	−0.09	−0.92	0.358	−11.67	4.25
FCGR2A rs1801274 (A/G)	0.48	2.00	0.03	0.24	0.812	−3.49	4.45
CASP3 rs113420705 (T/C)	−0.50	2.21	−0.03	−0.23	0.822	−4.89	3.90
Model 2 for IL-6 levels							
(Constant)	74.37	151.16		0.49	0.624	−225.16	373.89
ACE2 rs2074192 (C/T)	110.74	45.58	0.23	2.43	0.017^a^	20.42	201.06
IFNAR2 rs2236757 (G/A)	−50.51	49.83	−0.10	−1.01	0.313	−149.26	48.24
TYK2 rs2304256 (C/A)	0.89	50.44	0.01	0.02	0.986	−99.05	100.84
OAS1 rs10774671 (G/A)	−60.13	46.85	−0.12	−1.28	0.202	−152.96	32.70
OAS3 rs10735079 (A/G)	36.59	47.66	0.07	0.77	0.444	−57.85	131.02
CD40 rs4813003 (C/T)	−78.05	96.28	−0.07	−0.81	0.419	−268.83	112.73
FCGR2A rs1801274 (A/G)	−6.72	48.66	−0.01	−0.14	0.890	−103.13	89.70
CASP3 rs113420705 (T/C)	107.60	54.71	0.22	1.97	0.052	−0.81	216.01
Model 3 for IL-8 levels							
(Constant)	29.58	20.58		1.44	0.154	−11.25	70.42
ACE2 rs2074192 (C/T)	18.05	6.11	0.29	2.95	0.004^a^	5.93	30.18
IFNAR2 rs2236757 (G/A)	−9.54	6.51	−0.15	−1.47	0.146	−22.46	3.37
TYK2 rs2304256 (C/A)	1.11	6.60	0.02	0.17	0.866	−11.99	14.22
OAS1 rs10774671 (G/A)	6.21	6.26	0.10	0.99	0.324	−6.21	18.63
OAS3 rs10735079 (A/G)	−2.66	6.25	−0.04	−0.43	0.671	−15.06	9.74
CD40 rs4813003 (C/T)	−11.46	13.02	−0.08	−0.88	0.381	−37.28	14.37
FCGR2A rs1801274 (A/G)	−12.66	6.51	−0.20	−1.95	0.055	−25.57	0.25
CASP3 rs113420705 (T/C)	6.07	7.34	0.10	0.83	0.410	−8.50	20.63

^a^ Statistical significance.

**Table 6:** Impact of gene alleles on IL-12, TNF-α and IFN-α levels: regression results.

Predictors	Unstandardized coefficients	Standardized coefficients		t	*p*	95% confidence interval for B	
	B	Std. error	Beta			Lower bound	Upper bound
Model 4 for IL-12 levels							
(Constant)	98.07	46.80		2.10	0.039^a^	5.19	190.95
ACE2 rs2074192 (C/T)	−13.99	13.99	−0.10	−1.00	0.320	−41.76	13.78
IFNAR2 rs2236757 (G/A)	23.92	14.88	0.17	1.61	0.111	−5.61	53.44
TYK2 rs2304256 (C/A)	3.39	15.14	0.02	0.22	0.823	−26.66	33.45
OAS1 rs10774671 (G/A)	−6.18	14.36	−0.05	−0.43	0.668	−34.67	22.31
OAS3 rs10735079 (A/G)	−7.44	14.36	−0.05	−0.52	0.606	−35.94	21.06
CD40 rs4813003 (C/T)	−41.91	29.57	−0.14	−1.42	0.160	−100.59	16.78
FCGR2A rs1801274 (A/G)	−11.02	14.96	−0.08	−0.74	0.463	−40.72	18.68
CASP3 rs113420705 (T/C)	12.85	16.91	0.09	0.76	0.449	−20.71	46.40
Model 5 for TNF-α levels							
(Constant)	55.81	135.18		0.41	0.681	−212.05	323.67
ACE2 rs2074192 (C/T)	40.58	40.76	0.10	1.00	0.322	−40.19	121.35
IFNAR2 rs2236757 (G/A)	66.89	44.57	0.16	1.50	0.136	−21.42	155.20
TYK2 rs2304256 (C/A)	45.66	45.11	0.10	1.01	0.314	−43.72	135.04
OAS1 rs10774671 (G/A)	17.27	41.90	0.04	0.41	0.681	−65.75	100.29
OAS3 rs10735079 (A/G)	−15.05	42.62	−0.04	−0.35	0.725	−99.50	69.40
CD40 rs4813003 (C/T)	−60.37	86.10	−0.07	−0.70	0.485	−230.99	110.24
FCGR2A rs1801274 (A/G)	5.82	43.51	0.01	0.13	0.894	−80.40	92.04
CASP3 rs113420705 (T/C)	−47.77	48.93	−0.11	−0.98	0.331	−144.72	49.18
Model 6 for IFN-α levels							
(Constant)	75.87	25.63		2.96	0.004^a^	25.09	126.66
ACE2 rs2074192 (C/T)	−14.57	7.73	−0.17	−1.89	0.062	−29.88	0.74
IFNAR2 rs2236757 (G/A)	−21.64	8.45	−0.25	−2.56	0.012^a^	−38.38	−4.89
TYK2 rs2304256 (C/A)	−3.99	8.55	−0.04	−0.47	0.642	−20.93	12.96
OAS1 rs10774671 (G/A)	−17.22	7.94	−0.20	−2.17	0.032^a^	−32.96	−1.49
OAS3 rs10735079 (A/G)	14.48	8.08	0.16	1.79	0.076	−1.53	30.49
CD40 rs4813003 (C/T)	35.32	16.32	0.19	2.16	0.033^a^	2.98	67.66
FCGR2A rs1801274 (A/G)	−2.29	8.25	−0.03	−0.28	0.782	−18.64	14.05
CASP3 rs113420705 (T/C)	3.18	9.28	0.04	0.34	0.732	−15.20	21.56

^a^ Statistical significance.

**Table 7:** Model 1–6 summary results.

Model	Interleukin	R	*R* ^2^	Adjusted *R*^2^	Std. error of the estimate	F	*p*
Model 1	IL-1β	0.33	0.11	0.04	9.19	1.48	0.175
Model 2	IL-6	0.34	0.12	0.05	239.91	1.84	0.076
Model 3	IL-8	0.37	0.14	0.07	30.33	2.00	0.054
Model 4	IL-12	0.28	0.08	0.01	68.81	1.00	0.438
Model 5	TNF-α	0.22	0.05	−0.02	214.55	0.68	0.707
Model 6	IFN-α	0.41	0.17	0.11	40.67	2.83	0.007^a^

Predictors: Constant. ACE2 rs2074192 (alleles C/T). IFNAR2 rs2236757 (alleles G/A). TYK2 rs2304256 (alleles C/A). OAS1 rs10774671 (alleles G/A). OAS3 rs10735079 (alleles A/G). CD40 rs4813003 (alleles C/T). FCGR2A rs1801274 (alleles A/G). CASP3 rs113420705 (alleles T/C).

^a^ Statistical significance.

The overall model 1 for IL-1β, as summarised in Table 7, explains 11% of the variability in IL-1β levels. Despite the lack of statistical significance for Model 1, the individual regression coefficients revealed significant associations for two specific alleles. The *ACE2 rs2074192* variant showed a statistically significant positive association with IL-1β levels, indicating that the allele T is associated with a 3.92 unit increase in IL-1β levels. The *IFNAR2 rs2236757* variant also demonstrated a statistically significant positive association with IL-1β levels (B = 5.01, *p* = 0.017). However, the other genes did not exhibit statistically significant effects on IL-1β levels in the study population (*p* > 0.05).

Models 2 and 3 examined the effects of the same gene variants on IL-6 and IL-8 levels, respectively. The *ACE2 rs2074192* variant notably exhibited a highly significant positive association with IL-6 levels (B = 110.74, *p* = 0.017) and IL-8 levels (B = 18.05, *p* = 0.004).

In contrast, in Models 4 and 5, none of the studied genetic polymorphisms were significantly associated with IL-12 and TNF-α levels (*p* > 0.05).

Statistically significant Model 6 (*p* < 0.05) explains 17% of the variability of the IFN-α levels, nevertheless when eight predictors are involved only 11% of the variance in the dependent variable is explained by the current model. In Model 6, the *IFNAR2 rs2236757* allele A significantly negatively affected IFN-α levels (B = −21.64; *p* = 0.025, 95% CI: −40.51 to −2.76) as well as allele A *OAS1 rs10774671* gene (B = −17.22; *p* = 0.032, 95% CI: −32.96 to −1.49). At the same time, T allele *CD40 rs4813003* was related to the increase of IFN-α levels (B = 35.32; *p* = 0.033).

## Discussion

The analysis of cytokine profiles in pediatric patients with COVID-19, MIS-C, and healthy children revealed notable differences in levels of IL-1β, IL-6, IL-8, IL-12, TNF-α, and IFN-α levels. It is important to note the significant impact of age on immune response when discussing pediatric cohorts. The production of proinflammatory cytokines is directly correlated with age, while the secretion of anti-inflammatory cytokines is inversely related to the patient's age (21, 28). This may explain the stronger inflammatory response observed in the MIS-C patient cohort, as they were older than the COVID-19 group. Patients with MIS-C were found to have higher levels of proinflammatory cytokines compared to those with COVID-19. It is important to note that the timing of blood sampling in relation to the onset of symptoms and potential initiation of the cytokine storm differed significantly between the MIS-C and COVID-19 groups. This could be attributed to the evolution of the immune response, persistent immune activation, and prolonged inflammatory state, all of which play a significant role in the MIS-C manifestations. The cohort of children studied included patients with comorbidities that could potentially affect the levels of cytokines. However, due to the lack of significant differences in the frequency of comorbidities between children with MIS-C and COVID-19, as well as the small number of children with comorbidities in each study group (*n* = 4 and *n* = 2), the study did not analyze the impact of comorbidities on the interleukin profile of children. Therefore, our study primarily focused on the gene-interleukin associations without confounders.

To better understand the genetic patterns in children with COVID-19 and MIS-C, we conducted an analysis of gene polymorphism in individuals who were infected with SARS-CoV-2 (COVID-19 and MIS-C) and those who were not (healthy individuals). These findings were previously published and thoroughly assessed (2). However, with current study, we aimed to identify clinically important polymorphisms that may not only impact the course of COVID-19, but also influence on the secretion of interleukins, which could further determine COVID-19 progression.

Our study demonstrated an association between IL-1β, IL-6, IL-8, and TNF-α with the *ACE2 rs2074192* polymorphism, specifically with its T allele. This may be explained by the altered expression of ACE2 on the surface of cells. Thus, two factors in the pathogenesis of COVID-19 act complementarily−reduced ACE2 expression caused directly by the SARS-CoV-2 virus and the structural characteristics of the body's cells. As a result, the level of angiotensin II (Ang2), a potent pro-inflammatory modulator, increases. Through its interaction with neutrophils, mononuclear cells, and T and B cells, the production of IL-1β, IL-6, TNF-α, and IL-2 increases (12, 29). At the same time, Ang2, by binding to the AT1aR receptor, activates the enzymes ADAM10 and ADAM17, which in turn stimulate the production of soluble IL-6R (sIL-6R) as opposed to the formation of mIL-6R (membrane-bound IL-6 receptor). This process is accompanied by the release of soluble TNF-α (sTNFα) (30). This has important prognostic significance in COVID-19, as the binding of IL-6 to the formed sIL-6R leads to interaction with gp130 receptors, which are presented on many cells (immune cells, epithelial cells, fibroblasts). However, this cascade of interactions has limited regulation by SOCS3 (Suppressor of Cytokine Signaling 3), which leads to inflammation and the excessive release of pro-inflammatory cytokines (29).

The association of the *ACE2* gene polymorphism with IL-8 can also be explained by the specific binding of the SARS-CoV-2 virus through its S-protein (SP) to the ACE2 receptor, leading to the activation of the ERK1/2 (extracellular signal-regulated kinase 1/2) pathway (31). ERK1/2 is part of the MAPK (mitogen-activated protein kinase) signaling pathway, associated with the immune response, cell proliferation, and differentiation (31). Phosphorylated ERK1/2 activates transcriptional nuclear factors, particularly AP-1 (Activator Protein-1). AP-1 increases the expression of both IL-8 and IL-6 (31).

The results of our study demonstrated significantly lower IFN-α levels in carriers of the A allele of the *IFNAR2 rs2236757* gene. Despite a potential genetic predisposition, there are other factors that can influence the levels of IFN-α. This is evident in Model 6, where the Adjusted R2 value of 0.11 suggests that a majority of the variance can be attributed to predictors not included in the model with only eight genes. Previous studies have shown the antagonistic effect of SARS-CoV-2 on the interferon system, particularly on IFN-α production (32). Ten SARS-CoV-2 proteins have been identified that suppress IFN-α signaling and production: four nsp proteins (nsp1, nsp6, nsp7, and nsp13), four accessory proteins (ORF 3a, ORF 6, ORF 7a, and ORF 7b), and the virion M protein (8) (32–34). Some of them, specifically nsp6, nsp13, and ORF7b, inhibits the phosphorylation of the STAT1 and STAT2 signalling pathways, while ORF6 disrupts the nuclear translocation of STAT1 (32, 34). The M protein, through interaction with RIG-I-like receptors (RLR), has an inhibitory effect on the Mitochondrial Antiviral Signaling Protein (MAVS), consequently suppressing type I interferon secretion (32, 35). It is important to emphasize that up to 10% of patients with severe COVID-19 have autoantibodies to IFN-α, which neutralize high concentrations of type I interferons (11, 35). Therefore, it is important to consider both host genetic factors and the pathogenic role of the SARS-CoV-2 virus when predicting IFN-α levels in COVID-19 patients.

At the same time, a relationship between the *IFNAR2 rs2236757* gene polymorphism and the cytokines IL-1β and IL-12, with their levels increasing in the presence of the A allele. Previous studies have demonstrated that type I interferons reduce the secretion of IL-1β in two ways: by reducing the production of the pro-IL-1β protein and by inhibiting the activity of the NLRP1b and NLRP3 inflammasomes, which are necessary for the activation of caspase-1 and the subsequent proteolytic processing of pro-IL-1β (36–38). Specifically, tyrosine 701 phosphorylation of STAT-1 leads to suppressing the NLRP3 inflammasome. It should also be noted that the inhibitory effect of interferon on IL-1β can be mediated through the anti-inflammatory IL-10, which inhibits pro-IL-1β via the STAT-3 system (38). IL-10 is synthesized through the STAT1-mediated synergistic action of type I IFN and lipopolysaccharide (36, 37). It is important to emphasize that the reduced level of IFN-α has a suppressive effect on IL-12 secretion and also reduces the expression of IL-12R*β*2 on T cells (39). IFNAR2 is pathogenetically linked to Toll-Like Receptors (TLRs), a critical factor in regulating IL-12 synthesis. IFNAR2-mediated stimulation enhances the TLR-induced immune response, leading to an increase in IL-12p70 production (40).

Our research have not revealed any significant difference in interleukines level depending on the genetic polymorphism of *TYK2 rs2304256*. Nevertheless, the study of this gene is crucial in case of understanding of interleukin synthesis. The *TYK2 gene* encodes a non-receptor tyrosine kinase that is expressed in various immune system cells. TYK2 plays a crucial role in regulating intracellular signaling for many cytokines, including type I interferons (41, 42). Research indicates that the A allele of the *rs2304256* polymorphism in the *TYK2 gene* reduces the function of the tyrosine kinase, potentially decreasing the risk of developing autoimmune diseases (41). Additionally, *in vivo* studies have demonstrated that a deficiency in tyrosine kinase 2 leads to an absence of response to IFN-α (43). The expression of tyrosine kinase 2 on dendritic cells is an important factor in regulating the production of IL-12, which plays a key role in T cell activation and the formation of adaptive immune responses (44). Therefore, further research is necessary to uncover the potential connections between rs2304256 and cytokines in COVID-19, as well as in other diseases.

Our study established a significant difference in IFN-α levels depending on the polymorphism of the *OAS1 gene rs10774671*; however, when considering the combined influence of all eight genes, this gene did not show a statistically significant effect. Nevertheless, *OAS1 rs10774671* proved to be prognostically crucial for determining IL-8 levels in children infected with SARS-CoV-2. The expression of both OAS1 and OAS3 is regulated by the release of type I interferon, particularly IFN-α, under the influence of which ATP polymerization to 2'-5'-linked oligoadenylates (2–5A) occurs in the presence of viral RNA, ultimately leading to the formation of RNase L (45–47). Given the pathophysiological link between OAS1 and IFN-α, different genetic variants of the gene may form different protein isoforms with varying activity and, consequently, other responses to interferon signals. As a result, a feedback mechanism might lead to increased IFN-α synthesis. This could help compensate for the deficit of formed RNase L and maintain the immune response. However, in the context of our study, IFN-α levels were significantly lower in carriers of the A allele of *OAS1 rs10774671*, which could be explained by the generally low levels of IFN-α in children with COVID-19.

At the same time, the *OAS3* gene polymorphism *rs10735079* was associated with changes in IL-6 levels. Under conditions of altered OAS activity, RNase L-mediated degradation of SARS-CoV-2 RNA decreases. The increase in viral load stimulates pro-inflammatory cytokine synthesis, including IL-6. The obtained data also correlate with the results of Danyel Lee et al., which emphasize the enhancement of inflammatory processes in monocyte-lineage cells in the presence of genetic defects in OAS1, OAS2, or RNase L (48).

Patients carrying the C allele of the *CD40 gene rs4813003* in our study were associated with higher levels of IL-12 compared to carriers of the T allele of this gene. This association has a pathogenic basis. The CD40 receptor belongs to the Tumor Necrosis Factor Superfamily Receptors (TNFSFR) and is expressed on antigen-presenting cells (B cells, macrophages, dendritic cells) (49). The interaction between CD40 and CD40l, which is expressed in *T* cells, leads to the activation of Ras proteins (H-Ras and K-Ras), activating the p38 mitogen-activated protein kinase (MAPK) (50, 51). Activation of the p38 MAPK signalling pathway increases the transcription of two IL-12 subunits (IL-12p35 and IL-12p40) (51).

The T allele of the *CD40 gene rs4813003* was associated with an increase in IFN-α levels, as demonstrated by the regression analysis results. Increased expression of CD40 affects the STING (Stimulator of Interferon Genes)-mediated type I interferon (IFN-I) response, which plays a crucial role in early protection against infections (52, 53). Additionally, the interaction of CD40 with TRAF2/3 and TRAF6 (tumor necrosis factor receptor-associated factors) modulates STING ubiquitination, increasing their stability and supporting IFN-I production (52). The study by AbdelGhafar et al. demonstrates the relationship between CD40 gene polymorphism and the development of autoimmune diseases, as well as the production of specific cytokines, particularly the reduction of IFN-α levels (54).

The results of our study revealed a correlation between IL-8 levels and the *FCGR2A* gene polymorphism *rs1801274* (A/G). This gene encodes the Fc-gamma receptor IIa (FCGR2A), which serves as a receptor for the Fc fragment of immunoglobulin G (IgG) (55, 56). Binding of multiple IgG molecules to FCGR2A stimulates the immune system. Given that FCGR2A expression is quite widespread on the surface of many cells (monocytes, macrophages, neutrophils, dendritic cells, and platelets), the range of physiological responses to IgG interaction also varies−from stimulation of phagocytosis, antibody-dependent cellular cytotoxicity (ADCC), and the formation of reactive oxygen species (ROS) to the stimulation of cytokine production (55, 57). Previous studies have shown that neutrophils homozygous for AA (p.166His/His) exhibit stronger phagocytosis and degranulation in response to serum-opsonized bacteria compared to neutrophils homozygous for GG (p.166Arg/Arg) (55, 57). Because of degranulation, numerous inflammatory mediators, including IL-8, are released. Recent studies also demonstrate that cytokine synthesis is regulated by interacting with pattern recognition receptors (PRRs), such as Toll-like receptors (TLRs) and FCGRs, in response to an opsonized pathogen (56).

The *CASP3* gene polymorphism *rs113420705* led to a change in IL-6 levels, specifically an increase in IL-6, which may be associated with the activation of apoptosis and a rise in caspase-3 levels among carriers of the C allele. Research by Yildiz Gulhan et al. demonstrated that during SARS-CoV-2 invasion, the expression of CASP3 is altered to maintain homeostasis (58). The ORF3a, ORF6, ORF7a, M, N protein, and spike protein subunit 1 (S1) of SARS-CoV-2 stimulate the intrinsic or mitochondrial apoptotic pathway and activate caspase-3 (59–63). Activating caspase-3 can damage mitochondria and cause dysfunction, releasing mitochondrial DNA (mtDNA) and RNA (mtRNA). This leakage activates the cGAS-STING1 or RIG-I-MAVS pathways, which subsequently leads to the phosphorylation of TBK1 (TANK-binding kinase 1), the activation of transcription factors such as IRF3, and ultimately the synthesis of pro-inflammatory cytokines, particularly IL-6 (64). The role of Fas-mediated apoptosis in stimulating the production of both IL-6 and IL-8, as well as Monocyte Chemoattractant Protein-1 (MCP-1), is also emphasized (65).

Many of the gene variants studied in this research are located within the intronic regions of their corresponding genes. These include *rs2074192, rs2236757, rs10735079*, and *rs4813003*. The significance of investigating intronic gene variants lies in the fact that, despite being located in non-coding regions of DNA, they can still impact gene expression regulation, mRNA stability, and alternative splicing. This can result in the production of different protein isoforms, some of which may be non-functional or only partially functional (66). It is important to note that intronic variants are also associated with evolutionary changes, as their loss can lead to altered gene expression in an entire population (67). Furthermore, the role of intronic variants in clinical practice should not be overlooked, as they are believed to be key determinants of the genetic basis of diseases (66, 67). At the same time missense variant (*rs2304256, rs1801274*) could lead to the disruption in protein structure, stability or its function or do not have any impact on proteins (68). Nevertheless, their study is important in the context of disease appearance, cause missense mutations could lead to disease appearance (69). It is likely that the Splice Acceptor Variant (*rs10774671*) and the 5’ Untranslated Region (*rs113420705*) variant will be associated with changes in gene expression or impaired protein synthesis. This is expected to have a significant impact on the pathogenesis of disease.

Current literature suggests that the use of cytokine inhibitors, such as IL-1α and IL-1β receptor antagonists, anti-IL-6 monoclonal antibodies, anti-IL-6 receptor monoclonal antibodies, anti-TNF antibodies, and Janus kinase (JAK) inhibitors, is crucial therapeutic approach in treating COVID-19 (9) (70–72).

For MIS-C, current recommendations suggest using intravenous immunoglobulin and glucocorticoids as initial treatment for immunomodulation. When these treatments are ineffective, biological therapies like TNF inhibitors, IL-1 inhibitors, and IL-6 inhibitors may be considered (73, 74). However, the use of these inhibitors in pediatric practice is still limited, and their effectiveness is still being studied.

Our research provides new insight into potential strategies for managing MIS-C and severe COVID-19 in both children and adults, as genetic markers remain constant throughout life. This suggests that the effectiveness of COVID-19 treatment may vary based on an individual's genetic background.

The impact of the *ACE2 rs2074192* gene polymorphism on cytokine production (IL-1β, IL-6, IL-8, and TNF-α) is also significant and should be considered when implementing renin-angiotensin-aldosterone system targeting drugs in COVID-19 therapy (9, 75, 76). Furthermore, the influence of interferon-inducing genes (*IFNAR2 rs2236757, TYK2 rs2304256, OAS1 rs10774671, OAS3 rs10735079*) on cytokine production suggests the need for individualized use of interferon therapy protocols (77, 78).

Importantly, interpretation of obtained data could be restricted due to the small sample size, varying patient ages, varying time periods between disease progression and blood sample collection, as well as comorbidities presented. However, despite the small sample size due to the difficult wartime situation in Ukraine, our study has provided valuable insights into the role of gene polymorphism in the cytokine immune response and shed new light on this topic.

## Conclusion

Our research indicated that certain polymorphisms in the genes *ACE2 rs2074192, IFNAR2 rs2236757, OAS1 rs10774671, OAS3 rs10735079, CD40 rs4813003, FCGR2A rs1801274* and *CASP3 rs113420705* could predict cytokine responses in pediatric COVID-19 patients. These findings contribute to understanding the disease, developing targeted interventions for pediatric COVID-19, and providing personalized medical care for each patient.

The findings support the integration of genetic screening in clinical practice to stratify risk better and tailor therapeutic approaches for pediatric patients with COVID-19 and related inflammatory syndromes like MIS-C.

Our study further identifies genetic factors that affect cytokine responses, which could help address cytokine storms in COVID-19, particularly in children. This study is unique because it focuses on cytokines directly targeted by COVID-19 therapies, which could have implications for treatment in all age groups.

Additionally, by examining genes associated with Kawasaki disease (CD40, FCGR2A, CASP3), our study highlights potential common pathways in hyperinflammatory responses, suggesting that insights gained from COVID-19 could be applicable to other pediatric inflammatory conditions. Expanding our knowledge of cytokine immune responses about genetic predisposition and the correlation between genetic variations and specific cytokine responses indicates the possibility of developing personalized treatment strategies based on genetic profiling, which could significantly improve outcomes for pediatric COVID-19 patients.

Nevertheless, further studies with larger cohorts and more diverse cohorts are necessary to provide a more comprehensive understanding of the cytokine immune response, validate the associations we have found, and explore their clinical implications for pediatric COVID-19 and other viral infections.

## Data Availability

The datasets presented in this study can be found in online repositories. The names of the repository/repositories and accession number(s) can be found in the article/Supplementary Material.
